# Can We Apply WHOQOL-AGE to Asian Population? Verifying Its Factor Structure and Psychometric Properties in a Convenience Sample From Taiwan

**DOI:** 10.3389/fpubh.2020.575374

**Published:** 2020-11-24

**Authors:** Chung-Ying Lin, Jung-Der Wang, Li-Fan Liu

**Affiliations:** ^1^Institute of Allied Health Sciences, National Cheng Kung University Hospital, College of Medicine, National Cheng Kung University, Tainan, Taiwan; ^2^Department of Environmental and Occupational Health, College of Medicine, National Cheng Kung University, Tainan, Taiwan; ^3^Department of Internal Medicine, National Cheng Kung University Hospital, Tainan, Taiwan; ^4^Department of Public Health, College of Medicine, National Cheng Kung University, Tainan, Taiwan; ^5^Institute of Gerontology, College of Medicine, National Cheng Kung University, Tainan, Taiwan

**Keywords:** Asia, confirmatory factor analysis - CFA, elders, measurement invariance (MI), quality of life

## Abstract

**Objectives:** To translate and validate a recently developed quality of life instrument (WHOQOL-AGE) on geriatric population into Chinese.

**Method:** Using cross-sectional observational design, the WHOQOL-AGE was conducted among older people through interview. Confirmatory factor analysis (CFA) was used to examine the factor structure and multigroup CFA used to examine the measurement invariance.

**Results:** Through convenience sampling, 522 older adults (mean age = 73.42) participated in the study. Among them, 194 were males, 213 had an educational level at primary school or below, 398 were residing in the community, and 307 were aged 70 years or above. A bifactor structure (items Q1–Q8 are embedded in the factor 1; items Q9–Q13 embedded in the factor 2; and all the items embedded in an additional construct of QoL) was confirmed by the CFA in both the entire sample (χ^2^ = 25.4; df = 51; *p* = 0.999) and the subgroup sample with age 70 years or above (χ^2^ = 25.28; df = 51; *p* = 1.000). Multigroup CFAs results supported the measurement invariance for the WHOQOL-AGE across genders, having different educational levels, living in different settings and age groups. It also shows good known-groups validity.

**Conclusions:** The promising psychometric properties of the WHOQOL-AGE were found in our convenience sample of older Taiwanese. The supported measurement invariance indicates that the older people in different conditions of gender, educational level, and living setting interpret the WHOQOL-AGE similarly. However, our results should be interpreted with cautious because of the sample representativeness.

## Introduction

Due to modern medicine and improved technology, life expectancy has been extended worldwide (1). How to maintain quality of life (QoL), especially those aged over 65 years, has become a global concern (2). The World Health Organization (WHO) proposed the concept of QoL and developed corresponding measures in the 1990s (3). Specifically, QoL instruments are based on patient-reported outcomes (PROs), a primary outcome proposed by the U.S. Food and Drug Administration (4) to help healthcare professionals make clinical decisions using clients' feelings (5). With such unique characteristics, the number of QoL instruments has been growing in the literature, including those used in the elderly. Indeed, several generic QoL instruments have been verified as useful for assessing QoL among the older population (2, 6, 7).

Although generic QoL instruments have the advantage of comparing people with different conditions (8), some criticisms have been made. Taking the elderly population as an example, generic QoL instruments may not capture the most important aspects of QoL for the elderly (e.g., older people usually have worse hearing and vision than other adults, and this aspect is not included in the generic QoL instruments). Therefore, some researchers have developed more appropriate QoL instruments for the older population. For example, the Elderly Quality of Life Index (EQOLI) was developed in Brazil for longitudinal assessments of QoL change and potential impacts from behavior, intervention, and treatment (9, 10). The Quality of Life Scale for Elderly (QOLS-E) was developed in Japan using a sample of institutionalized people (11). The WHOQOL-OLD was developed as a supplementary module of the WHOQOL-BREF (12, 13).

However, a time concern has been raised for both the EQOLI and WHOQOL-OLD. Specifically, the EQOLI has 43 items, and the WHOQOL-OLD should be used together with the 26-item WHOQOL-BREF. Therefore, completing the EQOLI or WHOQOL-OLD plus WHOQOL-BREF may be a substantial burden for older people (14). Moreover, the QOLS-E was found to have somewhat low internal consistency (11). As a result, there is a need to develop a QoL instrument for older people with satisfactory psychometric properties.

In order to overcome the aforementioned problems, Caballero et al. (15) developed a stand-alone QoL instrument that assesses older people's QoL: WHOQOL-AGE. The 13-item WHOQOL-AGE was designed according to the characteristics of aging populations and was developed by integrating previous WHOQOL instruments, including the EUROHIS-QOL and the WHOQOL-OLD short form (12). The WHOQOL-AGE shows promising psychometric properties in nationally representative samples from Finland, Poland, and Spain (15). Moreover, older people from these three European countries somewhat interpreted the WHOQOL-AGE similarly (16). With good psychometric properties and a short administration time, the WHOQOL-AGE seems to be a feasible and appropriate instrument for use in large-scale population studies and busy clinical settings. Unfortunately, current literature shows that only two European studies (15, 16) have investigated the psychometric properties of the WHOQOL-AGE. Apart from the languages spoken in Finland, Poland, and Spain, the WHOQOL-AGE is not available in any other languages. Given that comparing QoL between people living in different countries requires the use of the same instrument, translating the WHOQOL-AGE into other languages is needed. Moreover, psychometric properties highly rely on the tested population; therefore, a sound instrument should be tested using different psychometric methods across different populations (8). If the requirements can be fulfilled, the psychometric evidence of the WHOQOL-AGE can be accumulated.

In addition to the lack of versions in other languages, the factor structure of the WHOQOL-AGE has not yet been confirmed, and the measurement invariance of the WHOQOL-AGE has never been examined across genders, educational levels, settings, and age groups. Although Santos et al. (16) applied confirmatory factor analysis (CFA) to examine whether the WHOQOL-AGE fits a two-factor model or a bifactor model better (for detailed information of the bifactor model, see Statistical Analysis section below), a model with cross-loading on item Q1 (How would you rate your quality of life?) found by Caballero et al. (15) was not compared. Santos et al. (16) found that the bifactor model fits better than the two-factor model and further demonstrated the partial invariance of the WHOQOL-AGE across countries in the bifactor model. However, it is unclear whether the WHOQOL-AGE has invariant factor structures across other important grouping factors. Specifically, gender, education, living context, and age usually impact an individual's cognition and, thus, results in different interpretations for the same item. Interpreting the items in a different way between groups may result in measurement bias between groups. Use education with the Q1 item (How would you rate your quality of life?) as an example. Those graduated from elementary school may think about basic needs (e.g., eating and living) when rating this item; those graduated from university may think about self-actualization (e.g., being respected). However, without the measurement invariance testing, we do not have the evidence to indicate whether the WHOQOL-AGE has the problem of measurement invariance in certain factors. Therefore, it is important to examine the measurement invariance of the WHOQOL-AGE across genders (male vs. female), educational levels (primary school or below vs. junior high school or above), living contexts or settings (community vs. institution, such as a nursing home), and age groups (below 70 vs. 70 years or above) after confirming its factor structure.

In order to fill the gap in the literature regarding the assessment of QoL among the elderly, the present study had the following aims. First, we aimed to translate the WHOQOL-AGE for an East Asian sample (i.e., Taiwanese). Second, to verify the factor structure of the WHOQOL-AGE among the Taiwanese elderly. After ensuring the factor structure of the WHOQOL-AGE, measurement invariance was examined to understand whether elderly people with different genders, educational levels, living settings, and ages interpret the WHOQOL-AGE differently.

## Methods

### Participants and Procedure

The main survey was conducted between October 2016 and March 2017. Participants were elderly persons aged 50 years and over who consented to be interviewed. Those who could not communicate (e.g., those with poor cognitive function or severe hearing impairment or who could not understand spoken Mandarin or Taiwanese) were excluded. Convenience sampling, mainly from the southern region of Taiwan (79%), was conducted to collect data from two groups: relatively healthy participants living in communities; patients or residents living in hospitals or long-term care (LTC) facilities with mild-to-severe dependency. All the participants were interviewed by experienced interviewers who fully understood the study aims. Interviews were conducted in the participants' homes, a quiet space nearby (for those living in the community), or the institution (for those living in an institution). The average time of each interview was about 10 min. Before collecting data, written informed consent was obtained from each participant. The study (A-ER-104-384) was approved by the Human Research Ethics Committee of National Cheng Kung University Hospital, and there were no conflicts of interest between the authors and the goals of this study. In total, 522 valid questionnaires were collected, including 398 (76.2%) from the communities and 124 (23.8%) from the institutions.

### Instruments: WHOQOL-AGE and Barthel Index

The WHOQOL-AGE contains 13 items and was developed from several European countries with nationally representative samples (15, 16). All the items are rated using a five-point Likert scale, the same scale that is used in the WHOQOL-BREF (17). Moreover, for the WHOQOL-AGE, items Q9–Q13, responses are classified as unipolar (e.g., *not at all* to *completely*) and for items Q1–Q8, responses are classified as bipolar (e.g., *very bad* to *very good*). Different factor structures have been proposed for the WHOQOL-AGE (for detailed information, please see Statistical Analysis section below), and the scoring method was proposed by Caballero et al. (15). In brief, a higher score indicates a better level of QoL. The psychometric properties of the WHOQOL-AGE have been verified in European countries: Cronbach's α = 0.84 to 0.91 (15); partial invariance across three European countries (Finland, Poland, and Spain) (16).

### Translation Procedure of the WHOQOL-AGE

Given that some WHOQOL-AGE items are identical to items in the WHOQOL-BREF and the Taiwan version of the WHOQOL-BREF has strong psychometric properties (17), those identical items were directly retrieved from the WHOQOL-BREF Taiwan version without translation. For the other WHOQOL-AGE items, they were first translated from English into Chinese by a bilingual translator and then back-translated into English by another bilingual translator. A bilingual expert in gerontology fine-tuned the Chinese WHOQOL-AGE (an interim version) after reviewing the forward translation, back translation, and the original English version of the WHOQOL-AGE. The interim version was then discussed and reviewed. The face-validity of the Chinese WHOQOL-AGE was confirmed by the experts committee.

### Statistical Analysis

Participants' characteristics were first analyzed using descriptive statistics. Independent *t*-tests and χ^2^ tests were used to compare the characteristics between participants living in the community and those living in an institution.

Four structural models were further tested using CFA. The four models included a one-factor model (Model 1), two two-factor models (Models 2 and 3), and a bifactor model (Model 4). Specifically, Model 1 had all items loaded on the same construct of QoL. Model 2, proposed by Santos et al. (16), had items Q1–Q8 embedded in Factor 1 and items Q9–Q13 embedded in Factor 2. Model 3, suggested by Caballero et al. (15), was a two-factor model with cross-loading on item Q1: items Q1–Q8 were embedded in Factor 1; items Q1 and Q9–Q13 were embedded in Factor 2. Model 4 was a bifactor model proposed by Santos et al. (16), in which items Q1–Q8 were embedded in Factor 1; items Q9–Q13 embedded in Factor 2, and all items embedded in an additional construct of QoL (Model 4).

The four models were examined using several fit indices to indicate whether the data fit these models. The fit indices included a χ^2^ test (in which a nonsignificant finding indicates fit), a comparative fit index (CFI; in which a value higher than 0.9 indicates fit), a Tucker-Lewis index (TLI; in which a value higher than 0.9 indicates fit), a standardized root mean square residual (SRMR; in which a value <0.08 indicates fit), and a root mean square error of approximation (RMSEA; in which a value <0.08 indicates fit) (18–20). Apart from the fit indices, the four models were compared using the χ^2^ difference test. Specifically, if a model had a significantly lower χ^2^ than another model, the former model had better fit (21). If some models had similar fits (i.e., no significant difference in the χ^2^ difference test), the simplest structure was viewed as the best model given the parsimony principle.

The best model determined using the χ^2^ difference test and the structure complexity was further used to examine measurement invariance across different conditions, including gender (male vs. female), educational level (≤elementary vs. ≥junior high), setting (community vs. institution), and age group (<70 vs. ≥70 years). Four sets of multigroup CFAs with nested models were applied to determine whether measurement invariance was supported across gender, educational level, setting, and age group. For each set of multigroup CFAs, there were three nested models, including a configural model, a model that constrained all the loadings to be equal between subgroups, and a model that constrained all the loadings and item intercepts to be equal between subgroups (22, 23). The three nested models were then compared using the χ^2^ difference test, the ΔCFI, and the ΔRMSEA. A nonsignificant χ^2^ indicated invariance across subgroups; however, the χ^2^ test is not recommended for use when sample size is large (i.e., *n* > 200) (18). Alternatively, ΔCFI > −0.01 and ΔRMSEA <0.01 also indicated invariance across subgroups (24, 25).

Because participants aged 70 years or above may have different perceptions on QoL from those aged below 70 years, we consider testing the WHOQOL-AGE only on those aged 70 years or above as a sensitivity analysis. The same sets of CFAs and multigroup CFAs were analyzed for the subgroup with age equal to or older than 70 years. However, multigroup CFAs on setting and age were not performed in the subgroup with age equal to or older than 70 years.

All the CFAs, including multigroup CFAs, were estimated using the diagonally weighted least square (DWLS) to tackle the Likert type responses in the WHOQOL-AGE (26). Cronbach's α and McDonald's ω were then applied to the confirmed structure of the WHOQOL-AGE to understand its internal consistency. A Cronbach's α > 0.7 and a McDonald's ω > 0.7 were considered as acceptable (26, 27). Moreover, known-group validity was tested to understand whether the WHOQOL-AGE could effectively distinguish the different levels of QoL between older people living in the community and those living in an institution. An independent *t*-test with effect size calculation (i.e., Cohen's *d*, where 0.2, 0.5, and 0.8 indicated small, moderate, and large effect size) was used for the known-group validity. According to our prior experience, interaction with the geriatric population, and the literature (28), we hypothesized that older people living in the community would have better QoL compared to those living in an institution.

All the analyses were done using the R software (R-3.5.1 for Windows). Additionally, CFAs and multigroup CFAs were performed using the lavaan package (http://lavaan.ugent.be/); Cronbach's α and McDonald's ω were calculated using the psych package (https://cran.r-project.org/web/packages/psych/index.html).

## Results

The mean age of the sample was 73.42 (SD = 10.46) years. Among the 522 participants, 194 (37.2%) were males, 213 (40.8%) had an educational level of primary school or below, and 398 (76.2%) were residing in a community. Additional demographic information is presented in Table 1. Moreover, Table 1 presents the characteristics of our participants who were aged 70 years or above (*N* = 307).

**Table 1:** Participants' characteristics.

**Characteristic**	**Total sample, *N* = 522**	**Sample aged over 70, *N* = 307**
**Age (years)**		
Mean (SD)	73.42 (10.76)	80.72 (6.78)
Range	50–105	70–105
**Age**		
Below 65 years	112 (21.5)	0 (0.0)
65 years and above	406 (77.8)	307 (100.0)
Missing	4 (0.7)	0 (0.0)
**Gender**, ***n*** **(%)**		
Male	194 (37.2)	131 (42.7)
Female	328 (62.8)	176 (57.3)
**Marital status**, ***n*** **(%)**		
Single	18 (3.4)	6 (2.0)
Married	299 (57.3)	140 (45.6)
Separated/Divorced	24 (4.6)	9 (2.9)
Widowed	177 (33.9)	151 (49.2)
Missing	4 (0.8)	1 (0.3)
**Education**, ***n*** **(%)**		
Primary school or below	213 (40.8)	169 (55.0)
Junior high school (9th grade) or above	305 (58.4)	138 (45.0)
Missing	4 (0.8)	0 (0.0)
**Living alone**, ***n*** **(%)**		
Yes	91 (17.4)	64 (20.8)
No	431 (82.6)	243 (79.2)
**Setting**, ***n*** **(%)**		
Community	398 (76.2)	209 (68.1)
Institution	124 (23.8)	98 (31.9)

All the proposed models (in both the entire and the subgroup samples) had satisfactory fit indices (CFI = 0.994–1.000, TLI = 0.993–1.009, SRMR = 0.029–0.060, and RMSEA = 0.000–0.029). Moreover, all the models had nonsignificant χ^2^ (*p* = 0.050–1.00), except for Model 1 in the entire sample (*p* = 0.016). When we used the χ^2^ difference test to compare the four models, Model 4 significantly outperformed Models 1 (*p* < 0.001), 2 (*p* < 0.001), and 3 (*p* < 0.001) in both the entire and the subgroup samples. Indeed, the fit indices of Model 4 were the best among all the proposed models (Table 2).

**Table 2:** Model comparisons.

**Model**	****χ^2^ (df)/***p***	**CFI^e^**	**TLI^e^**	**SRMR^e^**	**RMSEA^e^**	**90% CI for RMSEA**
**Entire sample**						
M1^a^	91.77 (65)/0.016	0.994	0.993	0.055	0.029	0.013, 0.042
M2^b^	82.97 (64)/0.056	0.996	0.995	0.052	0.024	0.000, 0.038
M3^c^	82.49 (63)/0.050	0.995	0.994	0.052	0.025	0.000, 0.039
M4^d^	25.40 (51)/0.999	1.000	1.009	0.029	0.000	0.000, 0.000
**Sample aged over 70 years**						
M1^a^	71.34 (65)/0.275	0.997	0.997	0.060	0.018	0.000, 0.041
M2^b^	67.26 (64)/0.366	0.999	0.998	0.059	0.013	0.000, 0.038
M3^c^	65.14 (63)/0.402	0.999	0.999	0.058	0.011	0.000, 0.037
M4^d^	25.28 (51)/0.999	1.000	1.016	0.035	0.000	0.000, 0.000

a *Model 1 is a one-factor model that all items loaded on the same construct (QoL)*.

b *Model 2 is a two-factor model proposed by Santos et al. (16): items Q1–Q8 in the factor 1; items Q9–Q13 in the factor 2*.

c *Model 3 is a two-factor model suggested by Caballero et al. (15): items Q1–Q8 in the factor 1; items Q1, Q9–Q13 in the factor 2*.

d *Model 4 is a bifactor model proposed by Santos et al. (16): items Q1–Q8 in the factor 1; items Q9–Q13 in the factor 2; all the items embedded in an additional construct of QoL*.

e *CFI, comparative fit index; TLI, Tucker-Lewis index; SRMR, standardized root mean square residual; RMSEA, root mean square error of approximation*.

Measurement invariance was examined using the best-fitting model among the four proposed models (i.e., Model 4). As shown in Table 3 (entire sample results) and Table 4 (results from those aged 70 years or above), the χ^2^ difference test showed no significant differences across gender between the configural model and the constrained loadings model (*p* = 0.42 and 0.57) and between the constrained loadings model and the constrained loadings and intercepts model (*p* = 0.68 and 0.92). Although significant differences were found between the configural model and the constrained loadings model across educational levels (*p* = 0.008 and 0.009), both ΔCFI (0.000) and ΔRMSEA (0.000) supported invariant loadings across educational levels and settings. Given that the χ^2^ test is sensitive to and easily significant in a large sample size (i.e., *n*>200), the ΔCFI and ΔRMSEA are the major indices to decide whether the measurement invariance is supported. The χ^2^ difference test showed no significant differences across educational levels between the constrained loadings model and the constrained loadings and intercepts model (*p* = 0.26 and 0.47). Furthermore, in the entire sample, no significant differences were found across settings and age groups between the configural model and the constrained loadings model (*p* = 0.07 and 0.23) and between the constrained loadings model and the constrained loadings and intercepts model (*p* = 0.80 and 0.99).

**Table 3:** Measurement invariance of bifactor model across gender, educational level, and setting using entire sample.

	**χ^2^ or (Δχ^2^)**	**df or (Δdf)**	***p*-value**	**CFI or (ΔCFI)^a^**	**RMSEA or (ΔRMSEA)^a^**
**Gender (male vs. female)**					
1. Configural	41.25	102	1.00	1.000	0.000
2. Loadings constrained	65.02	125	1.00	1.000	0.000
3. Loadings and intercepts constrained	72.51	135	0.98	1.000	0.000
2. vs. 1.	(23.77)	(23)	0.42	(0.000)	(0.000)
3. vs. 2.	(7.49)	(10)	0.68	(0.000)	(0.000)
**Educational level (≤elementary vs**. **≥junior high)**					
1. Configural	49.80	102	1.00	1.000	0.000
2. Loadings constrained	92.32	125	0.99	1.000	0.000
3. Loadings and intercepts constrained	104.67	135	0.98	1.000	0.000
2. vs. 1.	(42.52)	(23)	0.008	(0.000)	(0.000)
3. vs. 2.	(12.35)	(10)	0.26	(0.000)	(0.000)
**Setting (community vs. institution)**					
1. Configural	38.39	102	1.00	1.000	0.000
2. Loadings constrained	71.76	125	1.00	1.000	0.000
3. Loadings and intercepts constrained	77.91	135	1.00	1.000	0.000
2. vs. 1.	(33.37)	(23)	0.07	(0.000)	(0.000)
3. vs. 2.	(6.16)	(10)	0.80	(0.000)	(0.000)
**Age (< ** **70 years vs**. **≧** **70 years)**					
1. Configural	52.54	102	1.00	1.000	0.000
2. Loadings constrained	80.23	125	0.999	1.000	0.000
3. Loadings and intercepts constrained	82.48	135	1.00	1.000	0.000
2. vs. 1.	(27.69)	(23)	0.23	(0.000)	(0.000)
3. vs. 2.	(2.25)	(10)	0.99	(0.000)	(0.000)

a *CFI, comparative fit index; RMSEA, root mean square error of approximation*.

**Table 4:** Measurement invariance of bifactor model across gender, educational level, and setting using sample aged over 70 years.

	**χ^2^ or (Δχ^2^)**	**df or (Δdf)**	***p*-value**	**CFI or (ΔCFI)^a^**	**RMSEA or (ΔRMSEA)^a^**
**Gender (male vs. female)**					
1. Configural	41.96	102	1.00	1.000	0.000
2. Loadings constrained	63.22	125	1.00	1.000	0.000
3. Loadings and intercepts constrained	67.76	135	1.00	1.000	0.000
2. vs. 1.	(21.26)	(23)	0.57	(0.000)	(0.000)
3. vs. 2.	(4.54)	(10)	0.92	(0.000)	(0.000)
**Educational level (≤elementary vs**. **≥junior high)**					
1. Configural	40.06	102	1.00	1.000	0.000
2. Loadings constrained	82.06	125	0.999	1.000	0.000
3. Loadings and intercepts constrained	91.69	135	0.998	1.000	0.000
2. vs. 1.	(42.00)	(23)	0.009	(0.000)	(0.000)
3. vs. 2.	(9.63)	(10)	0.47	(0.000)	(0.000)

a *CFI, comparative fit index; RMSEA, root mean square error of approximation*.

Because the measurement invariance of the WHQOL-AGE was supported, we used the confirmed structure (i.e., Model 4) to test the internal consistency of the WHOQOL-AGE. Specifically, the entire WHOQOL-AGE and the two factors in the WHOQOL-AGE were examined using both Cronbach's α and McDonald's ω. Cronbach's α was 0.90 for the entire WHOQOL-AGE, 0.84 for Factor 1, and 0.81 for Factor 2. McDonald's ω was 0.91 for the entire WHOQOL-AGE, 0.88 for Factor 1, and 0.86 for Factor 2. The known-group validity further showed that older people living in the community had a better WHOQOL-AGE total score (Mean ± SD = 3.52 ± 0.57) than those living in an institution (Mean ± SD = 3.16 ± 0.63; *t* = 5.68; *p* < 0.001) with a moderate effect size (Cohen's *d* = 0.60).

## Discussion

With the rapid growth of the aging population (29), using validated instruments to assess the elderly's QoL is deemed to be important. From the perspective of healthy aging, reducing the possibilities of disease for older people can ease the caregiving burden for both society and family (29). A validated QoL instrument for older people can efficiently screen the health condition and provide timely and early intervention to prevent serious illnesses. Our study, thus, provides psychometric evidence of a brief and efficient QoL instrument specifically for use in the older population (i.e., WHOQOL-AGE) to echo the aforementioned needs. Moreover, our results show that the WHOQOL-AGE has promising construct validity (a bifactor structure) as verified by the satisfactory fit indices in CFA, and the WHOQOL-AGE had invariant factor structures across genders, educational levels, living settings, and ages.

To the best of our knowledge, only two studies (15, 16) have evaluated the psychometric properties of the WHOQOL-AGE prior to this study. Our results are comparable to those of the other two studies (15, 16). Specifically, satisfactory internal consistency for the WHOQOL-AGE was found in the study by Caballero et al. (15) as well as our study. Moreover, the satisfactory internal consistency was supported by different psychometric methods, including Cronbach's α [0.84–0.91 in Caballero et al.'s study (15); 0.81–0.90 in our study] and McDonald's ω (0.85–0.92 in our study).

Both our study and Santos et al.'s study (16) demonstrate that the bifactor model was the best fitting model with excellent fit for the WHOQOL-AGE. Therefore, we can ensure that the WHOQOL-AGE has promising construct validity and can assess global QoL by using all 13 items. Two factors were found in the WHOQOL-AGE, and Factor 1 items (i.e., Q1–Q8) seemed to share the *satisfaction in personal asset* concept while Factor 2 items (i.e., Q9–Q13) seemed to share the *self-efficacy in activities of daily living* concept. Nevertheless, Santos et al. (16) proposed another explanation for why there are two factors in the WHOQOL-AGE: The two factors were constructed because of their response scale (Factor 1 items are rated using bipolar response; Factor 2 items are rated using unipolar response). The different response scales can be considered as a method effect in the factor structure, and a similar type of method effect (e.g., positively and negatively worded items) has been illustrated in other QoL instruments (22, 30, 31). Therefore, the bifactor model verified that, after tackling the method effects, the WHOQOL-AGE can provide valid estimations of global QoL for older people.

We can further extend the findings from Santos et al.'s study (16) regarding the measurement invariance. Santos et al. (16) show the partial invariance of the WHOQOL-AGE across three European countries (Finland, Poland, and Spain). However, current literature provides no further information on the measurement invariance of the WHOQOL-AGE. Our study, thus, extends the findings of invariance across European countries to across genders, educational levels, living settings, and ages. Gender and educational level are obvious factors that may influence an individual to think differently and may lead to different interpretations of the same item descriptions (2). Therefore, it is important to evaluate whether the WHOQOL-AGE items are interpreted similarly across genders and educational levels. The supported measurement invariance found from our results indicated that neither gender nor educational level influenced the psychometric properties of the WHOQOL-AGE. Hence, the WHOQOL-AGE can be used to compare QoL between males and females and between those with low and high levels of education.

Moreover, apart from living in the community, older people can also live in unique settings; that is, LTC institutions or nursing homes. Given that the living context is different (32), older people may have different perceptions and considerations when they answer the items of the WHOQOL-AGE. Therefore, ensuring the measurement invariance of the WHOQOL-AGE across different living settings is important. The supported measurement invariance found from our results indicates that living context did not influence the psychometric properties of the WHOQOL-AGE. Hence, the WHOQOL-AGE can be used to compare QoL between those living in the community and those living in an institution.

As the measurement invariance was supported for the WHOQOL-AGE across different settings, we tested the known-group validity of the WHOQOL-AGE. Our results reveal consistent findings with the literature (28), which shows that older people living in the community had better QoL than those living in an institution. Moreover, our results on known-group validity showed that the WHOQOL-AGE had a moderate effect size in distinguishing QoL between different settings. Therefore, we anticipate that the WHOQOL-AGE would be a sensitive tool for detecting differences in QoL among older people. However, future studies may further investigate whether the WHOQOL-AGE is also sensible for detecting intervention effects.

There are some limitations in this study. First, we did not assess the test–retest reliability of the WHOQOL-AGE. Therefore, the reproducibility and stability of the WHOQOL-AGE remain unknown. Second, all the participants were recruited in Southern Taiwan through convenience sampling; thus, the representativeness is restricted, and the generalizability of our findings is limited. Third, although we tested the known-group validity of the WHOQOL-AGE using living settings, no other external criteria were assessed. Therefore, we are unsure whether the WHOQOL-AGE has satisfactory concurrent validity to support its underlying QoL concept. Future studies are, thus, warranted to investigate this topic using other validated QoL-related instruments (e.g., WHOQOL-BREF). Fourth, the present study did not examine the feasibility, reliability, and responsibility for the WHOQOL-AGE. Because a high-quality QoL instrument needs the information to potentiate its use, future studies are warranted to examine the feasibility, reliability, and responsibility of the WHOQOL-AGE in Asia. Last and importantly, although we have ensured the linguistic validity of the Taiwan version WHOQOL-AGE during the translation process, we did not design culturally specific items for the Taiwan version. Given that the WHOQOL-AGE was developed from European countries and that European and Taiwanese lifestyles are different, the translated WHOQOL-AGE may not be able to detect the QoL for Taiwanese people specifically. Future studies may consider developing and incorporating culturally specific items into the Taiwan version WHOQOL-AGE.

## Conclusions

Our findings may supplement the use of QoL on older people in the current literature. Specifically, WHOQOL-OLD is a well-established instrument with strong psychometric properties to assess QoL for older people, and our psychometric findings on WHOQOL-AGE may provide healthcare providers another choice to assess QoL for older people. That is, we may consider using them in different situations. Specifically, the WHOQOL-OLD has more items than does the WHOQOL-AGE; therefore, WHOQOL-OLD can provide more detailed QoL information than does the WHOQOL-AGE. Thus, the WHOQOL-OLD is a good instrument when a user wants to obtain detailed QoL information for older people. In contrast, the WHOQOL-AGE has fewer items and, therefore, can be used when a user wants to quickly obtain the QoL information of older people.

In conclusion, the present study demonstrates the promising psychometric properties of the WHOQOL-AGE in an East Asian sample through convenience sampling. With the strong psychometric properties found in this study, other Asian countries may consider translating the WHOQOL-AGE and examine whether it can efficiently assess QoL for the elderly. Moreover, the supported measurement invariance of the WHOQOL-AGE indicates that it can precisely assess QoL for older people in different conditions, including different genders, educational levels, and living settings. Nevertheless, future studies should consider using a representative sample to examine the psychometric properties of the WHOQOL-AGE (including test–retest reliability and concurrent validity that were not tested in the present study) to gather additional information to corroborate the usefulness of the WHOQOL-AGE in Asia. Specifically, results from the present study can only be generalized to a small portion of Taiwanese older people and additional evidence is needed.

## Data Availability Statement

The raw data supporting the conclusions of this article will be made available by the authors, without undue reservation.

## Ethics Statement

The studies involving human participants were reviewed and approved by Human Research Ethics Committee of National Cheng Kung University Hospital (A-ER-104-384), and no conflicts of interest between the authors and the goals of this study. The patients/participants provided their written informed consent to participate in this study. Written informed consent was obtained from the individual(s) for the publication of any potentially identifiable images or data included in this article.

## Author Contributions

C-YL: data analyses, preparation of the manuscript. J-DW: study design, validation of the WHOQOL-AGE translation version, proof reading of manuscript. L-FL: design of the research, fund raising, data collection, and preparation of the manuscript. All authors contributed to the article and approved the submitted version.

## Conflict of Interest

The authors declare that the research was conducted in the absence of any commercial or financial relationships that could be construed as a potential conflict of interest.

## References

[1] Lee R Mortality forecasts and linear life expectancy trends. In: Bengtsson T Keilman N editor. Old and New Perspectives on Mortality Forecasting. Demographic Research Monographs. New York, NY: Springer (2019). p. 167–83.

[2] Lin C-Y Li Y-P Lin S-I Chen C-H. Measurement equivalence across gender and education in the WHOQOL-BREF for community-dwelling elderly Taiwanese. Int Psychogeriatr. (2016) 28:1375–82. 10.1017/S1041610216000594 27097756

[3] The WHOQOL Group. Development of the World Health Organization WHOQOL-BREF quality of life assessment. Psychol Med. (1998) 28:551–8. 10.1017/S0033291798006667 9626712

[4] Food and Drug Administration Guidance for Industry—Patient-Reported Outcome Measures: Use in Medical Product Development to Support Labeling Claims. Silver Spring, MD: FDA (2009). 43 p.

[5] Chang KC Wang JD Tang HP Cheng CM Lin CY. Psychometric evaluation using Rasch analysis of the WHOQOL-BREF in heroin-dependent people undergoing methadone maintenance treatment: further item validation. Health Qual Life Outcomes. (2014) 12:148. 10.1186/s12955-014-0148-6 25277717 PMC4190329

[6] Hayes V Morris J Wolfe C Morgan M. The SF-36 health survey questionnaire: is it suitable for use with older adults? Age Ageing. (1995) 24:120–5. 10.1093/ageing/24.2.120 7793333

[7] Wolfs CA Dirksen CD Kessels A Willems DC Verhey FR Severens JL. Performance of the EQ-5D and the EQ-5D+C in elderly patients with cognitive impairments. Health Qual Life Outcomes. (2007) 5:33. 10.1186/1477-7525-5-33 17570832 PMC1904437

[8] Lin C-Y Hwang J-S Wang W-C Lai W-W Su W-C Wu T-Y. Psychometric evaluation of the WHOQOL-BREF, Taiwan version, across five kinds of Taiwanese cancer survivors: Rasch analysis and confirmatory factor analysis. Taiwan Yi Hxu Hui Za Zhi. (2109) 118:215–22. 10.1016/j.jfma.2018.03.018 29661488

[9] Paschoal SM Filho WJ Litvoc J. Development of elderly quality of life index - EQOLI: theoretical-conceptual framework, chosen methodology, and relevant items generation. Clinics. (2007) 62:279–88. 10.1590/S1807-59322007000300012 17589668

[10] Paschoal SM Jacob FW Litvoc J. Development of elderly quality of life index - EqoLI: item reduction and distribution into dimensions. Clinics. (2008) 63:179–88. 10.1590/S1807-59322008000200005 18438571 PMC2664201

[11] Hoshino K Yamada H Endo H Nagura E. An preliminary study on quality of life scale for elderly: an examination in terms of psychological satisfaction. Shinrigaku Kenkyu. (1996) 67:134–40. 10.4992/jjpsy.67.134 8829293

[12] Fang J Power M Lin Y Zhang J Hao Y Chatterji S. Development of short versions for the WHOQOL-OLD module. Gerontologist. (2012) 52:66–78. 10.1093/geront/gnr085 22021402

[13] Power M Quinn K Schmidt S. Development of the WHOQOL-old module. Qual Life Res. (2005) 14:2197–214. 10.1007/s11136-005-7380-9 16328900

[14] Friedman B Heisel MJ Delavan RL. Psychometric properties of the 15-item geriatric depression scale in functionally impaired, cognitively intact, community-dwelling elderly primary care patients. J Am Geriatr Soc. (2005) 53:1570–6. 10.1111/j.1532-5415.2005.53461.x 16137289

[15] Caballero FF Miret M Power M Chatterji S Tobiasz-Adamczyk B Koskinen S. Validation of an instrument to evaluate quality of life in the aging population: WHOQOL-AGE. Health Qual Life Outcomes. (2013) 11:1–12. 10.1186/1477-7525-11-177 24152691 PMC4015924

[16] Santos D Abad FJ Miret M Chatterji S Olaya B Zawisza K. Measurement invariance of the WHOQOL-AGE questionnaire across three European countries. Qual Life Res. (2018) 27:1015–25. 10.1007/s11136-017-1737-8 29143905

[17] Yao G Chung C-W Yu C-F Wang J-D. Development and verification of validity and reliability of the WHOQOL-BREF Taiwan version. Taiwan Yi Xue Za Zhi. (2002) 101:342–51. 12101852

[18] Kyriazos T Applied psychometrics: sample size and sample power considerations in factor analysis (EFA, CFA) and SEM in general. Psychology. (2018) 9:2207–30. 10.4236/psych.2018.98126

[19] Fung XCC Pakpour AH Wu K-Y Fan C-W Lin C-Y Tsang HHW. Psychosocial variables related to weight-related self-stigma in physical activity among young adults across weight status. Int J Environ Res Public Health. (2019) 17:64. 10.3390/ijerph17010064 31861769 PMC6981798

[20] Lin C-Y. Comparing quality of life instruments: sizing them up vs. PedsQL and Kid-KINDL. Soc Health Behav. (2018) 1:42–7. 10.4103/SHB.SHB_25_1830619497

[21] Chang C-C Lin C-Y Gronholm PC Wu T-H. Cross-validation of two commonly used self-stigma measures, Taiwan versions of the internalized stigma mental illness scale and self-stigma scale-short, for people with mental illness. Assessment. (2018) 25:777–92. 10.1177/1073191116658547 27385391

[22] Lin C-Y Strong C Tsai M-C Lee C-T. Raters interpret positively and negatively worded items similarly in a quality of life instrument for children: Kid-KINDL. Inquiry. (2017) 54:1–7. 10.1177/0046958017696724 28292193 PMC5798710

[23] Lin C-Y Imani V Cheung P Pakpour AH. Psychometric testing on two weight stigma instruments in Iran: weight self-stigma questionnaire and weight bias internalized scale. Eat Weight Disord. (2020) 25:889–901. 10.1007/s40519-019-00699-4 31055783

[24] Leung H Pakpour AH Strong C Lin Y-C Tsai M-C Griffiths MD. Measurement invariance across young adults from Hong Kong and Taiwan among three internet-related addiction scales: Bergen Social Media Addiction Scale (BSMAS), Smartphone Application-Based Addiction Scale (SABAS), and Internet Gaming Disorder Scale-Short Form (IGDS-SF9)(Study Part A). Addict Behav. (2020) 101:105969. 10.1016/j.addbeh.2019.04.027 31078344

[25] Pakpour AH Tsai M-C Lin Y-C Strong C Latner JD Fung XCC. Psychometric properties and measurement invariance of the Weight Self-Stigma Questionnaire and Weight Bias Internalization Scale in Hongkongese children and adolescents. Int J Clin Health Psychol. (2019) 19:150–9. 10.1016/j.ijchp.2019.03.001 31193103 PMC6517648

[26] Lin C-Y Pakpour AH Broström A Fridlund B Årestedt K Strömberg A. Psychometric properties of the 9-item European Heart Failure self-care behavior scale using confirmatory factor analysis and rasch analysis among Iranian patients. J Cardiovasc Nurs. (2018) 33:281–8. 10.1097/JCN.0000000000000444 28858887

[27] Hou W-L Lin C-Y Wang Y-M Tseng Y-H Shu B-C. Assessing related factors of intention to perpetrate dating violence among university students using the theory of planned behavior. Int J Environ Res Public Health. (2020) 17:923. 10.3390/ijerph17030923 32024264 PMC7036797

[28] Karakaya M Bilgin S Ekici G Köse N Otman A. Functional mobility, depressive symptoms, level of independence, and quality of life of the elderly living at home and in the nursing home. J Am Med Dir Assoc. (2009) 10:662–6. 10.1016/j.jamda.2009.06.002 19883891

[29] Hess M Nauman E Steinkopf L Population ageing, the intergenerational conflict, and active ageing policies – a multilevel study of 27 European Countries. J Pop Ageing. (2017) 10:11–23. 10.1007/s12062-016-9161-3

[30] Lee C-T Lin C-Y Tsai M-C Strong C Lin Y-C. Psychometric evaluation and wording effects on the Chinese version of the parent-proxy Kid-KINDL. Health Qual Life Octcomes. (2016) 14:123. 10.1186/s12955-016-0526-3 27595602 PMC5011907

[31] Lin C-Y Luh W-M Cheng C-P Yang A-L Ma H-I Evaluating the wording effect and psychometric properties of the Kid-KINDL: using the multitrait-multimethod approach. Eur J Psychol Assess. (2014) 30:100–9. 10.1027/1015-5759/a000175

[32] Coward RT Netzer JK Mullens RA. Residential differences in the incidence of nursing home admissions across a six-year period. J Gerontol B Psychol Sci Soc Sci. (1996) 51:S258–67. 10.1093/geronb/51B.5.S258 8809011

