# Supplementary material for: Can We Apply WHOQOL-AGE to Asian Population? Verifying Its Factor Structure and Psychometric Properties in a Convenience Sample From Taiwan
Source: Front Public Health. 2020 Nov 24;8:575374. doi: 10.3389/fpubh.2020.575374 (PMC7732621; doi:10.3389/fpubh.2020.575374)
Supplement: Supplementary file 1 [file Data_Sheet_1.docx]

Appendix: Chinese Version of WHOQOL-AGE

**台灣版世界衛生組織生活品質跨世代問卷(WHOQOL-AGE)**

此份問卷詢問您對生活品質方面的想法及感覺，如果對於回答不確定，請選出五個答案中最適合的，通常為最早想到的那個答案。本問卷關心的是您最近的生活情況，請您以自己的標準來回答下列問題。接著，請您仔細閱讀下列13道題目，評估您自己的感覺，並選出最適合您的答案於□內打「🗸」。

誠摯感謝您的協助！

1. 整體來說，您如何評價您的生活品質？

□極不好　 □不好　 □中等程度好　 □好 □極好

2. 整體來說，您滿意自己的聽力、視力或其他感官功能嗎？

□極不滿意 □不滿意 □中等程度滿意 □滿意 □極滿意

3. 整體來說，您滿意自己的健康嗎？

□極不滿意 □不滿意 □中等程度滿意 □滿意 □極滿意

4. 整體來說，您對自己滿意嗎？

□極不滿意 □不滿意 □中等程度滿意 □滿意 □極滿意

5. 您滿意自己從事日常活動的能力嗎？

□極不滿意 □不滿意 □中等程度滿意 □滿意 □極滿意

6. 您滿意自己的人際關係嗎？

□極不滿意 □不滿意 □中等程度滿意 □滿意 □極滿意

7. 您滿意自己住所的狀況嗎？

□極不滿意 □不滿意 □中等程度滿意 □滿意 □極滿意

8. 您滿意自己時間利用的情況嗎？

□極不滿意 □不滿意 □中等程度滿意 □滿意 □極滿意

9. 您每天的生活有足夠的精力嗎？

□完全不足夠 □少許足夠 □中等程度足夠 □很足夠 □完全足夠

10. 您能夠掌控想做的事情嗎？

□完全不能夠 □有一點能夠 □中等程度能夠 □很能夠 □極能夠

11. 您對有機會繼續實現生活目標的滿意程度如何？

□完全不滿意 □少許滿意 □中等程度滿意 □很滿意 □完全滿意

12. 您有足夠的金錢應付所需嗎?

□完全不足夠 □少許足夠 □中等程度足夠 □很足夠 □完全足夠

13. 您滿意自己生活中的親密關係嗎?

□極不滿意 □不滿意 □中等程度滿意 □滿意 □極滿意

**問卷到此結束，感謝您的填答！**
